# A Review of Cardiac Manifestations in Patients With Systemic Lupus Erythematosus and Antiphospholipid Syndrome With Focus on Endocarditis

**DOI:** 10.7759/cureus.21698

**Published:** 2022-01-28

**Authors:** Matthew G Tayem, Linda Shahin, John Shook, Marc M Kesselman

**Affiliations:** 1 Internal Medicine, Nova Southeastern University Dr. Kiran C. Patel College Of Osteopathic Medicine, Davie, USA; 2 College of Dentistry, Nova Southeastern University, Davie, USA; 3 Rheumatology, Nova Southeastern University Dr. Kiran C. Patel College Of Osteopathic Medicine, Davie, USA

**Keywords:** endocarditis, systemic lupus erythematosus, sle, non-bacterial thrombotic endocarditis, nbte, antiphospholipid syndrome, autoimmune diseases and endocarditis, autoimmune diseases, aps, libman-sacks endocarditis

## Abstract

Patients with autoimmune diseases such as systemic lupus erythematosus (SLE) or antiphospholipid syndrome (APS) are at a higher risk for adverse cardiovascular events associated with increased morbidity and mortality. The increased risk of these events is often associated with rheumatic heart disease (heart valve or mural endocardium damage from rheumatic fever) following microbial infection (i.e., untreated or under-treated streptococcal infection). In particular, the weakening of cardiac vasculature due to rheumatic heart disease makes such patients with autoimmune diseases more susceptible to endocarditis. Endocarditis can be caused by an infection (infective endocarditis) or inflammation tied to disease activity (non-bacterial thrombotic endocarditis [NBTE]). Infective endocarditis among patients with autoimmune diseases may result from exposure to pathogens during dental or surgical procedures. NBTE commonly occurs as a result of fibrin and platelet aggregation on the cardiac valves without bacterial infection. While diagnosis and management can vary based on underlying etiology, an interdisciplinary approach that includes prevention and management from dentists, cardiologists, rheumatologists, and primary care physicians is needed. In addition, increasing patient and physician education on risk factors and prevention strategies is much needed. This manuscript will review the pathophysiology of endocarditis, the association between SLE and APS and endocarditis risk, the diagnosis and management of these autoimmune diseases with a focus on the prevention of cardiovascular disease risk, and make recommendations for diagnostic and management approaches to improve care.

## Introduction and background

Pathophysiology of endocarditis

**Figure 1:**
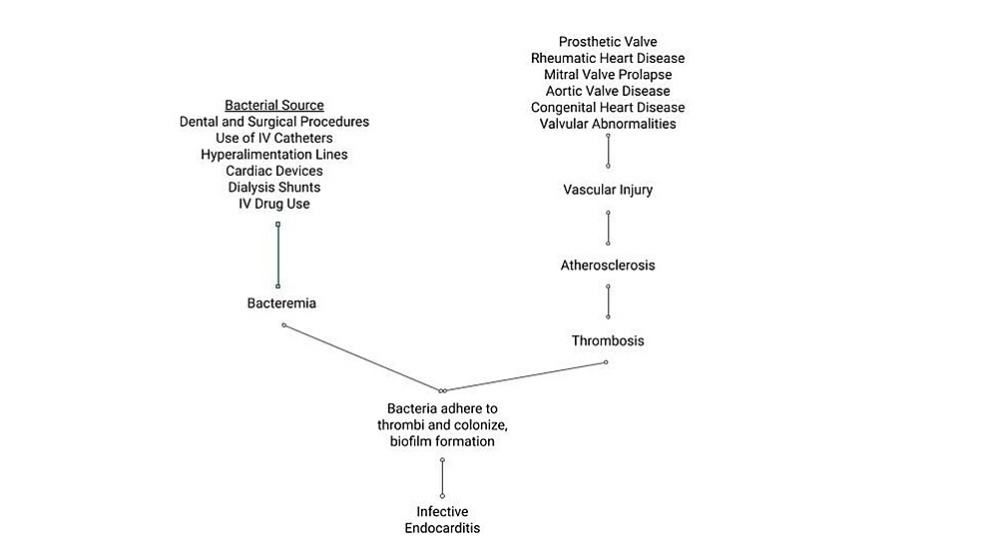
Proposed mechanisms of the pathophysiology of infective endocarditis

Endocarditis is the inflammation of the inner lining of the heart, the endocardium, as well as the valves of the heart. It is primarily caused by bacterial infection of the heart and, in such cases, is called infective endocarditis (IE). As proposed in Figure 1, IE can result from infection occurring during dental and surgical procedures, from the use of intravascular catheters, hyperalimentation lines, cardiac devices, and dialysis shunts, as well as from intravenous drug use. Streptococci and Staphylococci account for approximately 80 percent of all IE cases. Enterococci is the third leading cause of IE and is linked to healthcare interventions. Gram-negative and fungal microorganisms are rare in IE infections [1]. Without early detection and treatment, a wide array of cardiac complications can develop. It is most prevalent among males over 50 years old and, depending on the pathogen, such as *Staphylococcus aureus*, is associated with high morbidity and mortality rates of greater than 25 percent even after therapy [2,3]. Meanwhile, careful evaluation and detection helps physicians diagnose and manage the disease, limiting mortality and morbidity.

Another form of endocarditis that is less prevalent is non-bacterial thrombotic endocarditis (NBTE), which involves fibrin and platelet aggregation on the cardiac valves without bacteremia [4]. NBTE can occur due to mechanical stress, chemical agents, and from immunological conditions (autoimmune diseases) such as systemic lupus erythematosus (SLE) and antiphospholipid syndrome (APS). Blood culture-negative endocarditis accounts for 2.5 percent to 31 percent of all cases of endocarditis [5]. Although uncommon, it is frequently underestimated in the clinical setting due to its atypical presentation. 

NBTE can be a potentially life-threatening source of thromboembolism, resulting in infarction to vital organs such as the brain and heart. Factors associated with NBTE include hypoxia, immune complex formation, and hypercoagulability, which lead to endothelial damage and subsequent exposure to circulating platelets. While histopathology studies have described NBTE as fibrin buildup with plasma cells and lymphocytes on the mitral valve, the condition is not always recognized on echocardiographic images such as transthoracic echocardiography (TTE) compared to the more sensitive option of transesophageal echocardiography (TEE). Clinicians are urged to perform a patient workup that involves identification of a hypercoagulable state (determination of the presence of lupus anticoagulant, elevated levels of antiphospholipid antibodies, disseminated intravascular coagulation [DIC]), and presence of malignancy [4]. 

The diagnosis of endocarditis involves screening of presenting symptoms, medical history (including surgical, dental, and/or invasive procedure history), physical examination, and laboratory testing. Clinically, IE and NBTE have similar signs and symptoms. The diagnostic approaches that are helpful in identifying IE include a comprehensive history, physical examination, and laboratory tests for inflammatory markers, such as rheumatoid factor (RF) and antinuclear antibodies [6]. In addition, the Duke criteria, which is a set of clinical criteria used for the diagnosis of IE, is commonly used. The Duke criteria includes major criteria (positive blood cultures for infective endocarditis, presence of typical microorganisms on two blood cultures, and evidence of endocardial involvement) and minor criteria (present with a fever greater than 38°C, predisposing heart condition or intravenous drug use, vascular and/or immunogenic phenomena, microbiologic findings, and echocardiographic findings indicative of infective endocarditis), with diagnosis based on patients meeting two major and one minor criterium, one major and three minor criterium, or five minor criterium [1,7]. The diagnosis of NBTE involves history and physical examination, focus on cardiac manifestations, echocardiography, and computer tomography. 

In this manuscript, we will review the evidence for correlations between SLE and APS and endocarditis disease risk. Several studies have highlighted the roles various autoimmune diseases, SLE and APS, play in the development of cardiovascular disease, specifically endocarditis. Autoimmune disease states, such as rheumatic carditis, may alter the cardiac valve surface as a result of factors such as turbulent blood flow [1,8]. Most commonly, rheumatic heart disease, a condition characterized by heart valve or mural endocardium damage due to rheumatic fever following microbial infection (untreated or under-treated streptococcal infection), is associated with the formation of vegetations composed of thrombotic debris and organisms, leading to the destruction of the underlying heart tissue [2,8]. As a result, the condition may make autoimmune patients more susceptible to endocarditis.

## Review

Endocarditis and systemic lupus erythematosus (SLE)

SLE causes systemic inflammation within the connective tissue of any organ system (including the cardiovascular system) and most commonly impacts women. Cardiac involvement in patients with SLE can negatively impact all components of the cardiovascular system and heart, including the pericardium, conducting system, myocardium, valves, and coronary arteries, and is associated with increased morbidity and mortality [9]. The prevalence of coronary heart disease in patients with SLE ranges from 6 to 10 percent [10]. The clinical manifestations of coronary artery disease (CAD) in SLE can be attributed to various pathophysiologic mechanisms, including atherosclerosis and thrombosis [9]. Atherosclerosis does not only occur more frequently in SLE patients compared to the general population, but it is also accelerated. In a cross-sectional study, plaque was present in 37 percent of patients with SLE as compared with 15 percent of healthy controls [10]. Inflammation is central to the pathogenesis of cardiovascular disease in SLE patients. In addition, autoantibodies and the development of immune complexes with complement activation play a significant role in cardiovascular injury among SLE patients. While many traditional risk factors for CAD in SLE patients include hyperlipidemia, diabetes, smoking, obesity, and hypertension, other nontraditional risk factors include renal disease and corticosteroid use [9]. Modification of these risk factors should be prioritized in these patients.

Libman-Sacks endocarditis (LSE), which is a type of NBTE, can be associated with cardiac manifestations among SLE patients. Libman-Sacks valve lesions are microscopically characterized by fibrin deposits at various fibroblastic organization and neovascularization stages and by a variable extent of inflammation with mononuclear cell infiltration. LSE is a rare disease found chiefly post-mortem, with an approximate prevalence between 0.9 and 1.6 percent. Libman-Sacks commonly affects SLE patients between 40 and 80 years of age. SLE is most common among women of childbearing age as compared to men and more prevalent among Black and Hispanic women than White women. Pathological studies reveal various underlying damage in SLE patients that predispose them to Libman-Sacks, including vegetations on the mitral and aortic valve surfaces, chordae tendineae, papillary muscles, and mural endocardium. In addition, the presence of lumen sacs with interstitial edema, fibrosis, and chronic inflammation of the myocardium may be seen in these immune-deficient patients [4]. The etiology of NBTE is poorly understood but thought to be attributed to a combination of different interacting mechanisms such as circulating immune complexes in autoimmune disease patients, which leads to a hypercoagulable state and potential valvular destruction. Due to the presence of Libman-Sacks lesions, SLE patients have an increased frequency of functionally impaired cardiac valves, which places them at a higher risk of developing infective endocarditis as well [11]. 

To diagnose LSE, a high level of clinical acumen is needed. While a diagnosis can be established pathologically through autopsy or surgical intervention of platelet thrombi, there is no laboratory test that can confirm the diagnosis of Libman-Sacks endocarditis [4]. Still, using echocardiography such as transesophageal echocardiography, histological findings, and immunological tests, a proper diagnosis can be made with high confidence. Echocardiography is the primary method of evaluation for LSE; for example, transesophageal echocardiography is 80 to 90 percent more sensitive than transthoracic echocardiography. To differentiate LSE from IE, complete blood and metabolic panel, as well as blood cultures, need to be assessed. In addition, a hypercoagulable workup, which includes lupus anticoagulant and antiphospholipid antibodies, is necessary for diagnosis [4]. The absence of typical microorganisms, which cause IE, can provide support for the diagnosis of non-bacterial thrombotic embolism. 

There currently are no randomized, controlled trials that demonstrate the effectiveness of cardiovascular (CV) interventions associated with SLE. As such, treatment recommendations for SLE patients with CV risk follow recommendations for other at-risk populations. The underlying pathophysiology of SLE requires treatment initially. Recommendations include lifestyle modifications such as smoking cessation, regular aerobic exercises, and maintaining a normal body mass index (BMI). In addition, statins are recommended for SLE patients to lower low-density lipoprotein (LDL) cholesterol levels below 100 mg/dl. Furthermore, angiotensin-converting enzyme (ACE) inhibitors are the first choice in treating hypertension to maintain a blood pressure of less than 130/80 mmHg [12]. Unless contraindicated, the use of low-dose aspirin daily is recommended for patients with SLE. Research also suggests that corticosteroid use should be minimized early in the disease. Corticosteroids significantly increased the risk of CV events in patients with SLE. According to an autopsy study, more than a 50 percent narrowing by atherosclerotic plaque was seen in one of the main coronary vessels in 42 percent of patients who were treated with steroids over a one-year period [12]. Anticoagulation therapy is considered for secondary prevention for thromboembolic events in patients who have experienced a prior event. Meanwhile, patients with LSE should be monitored closely during anticoagulation therapy, as they are at a higher risk of developing thromboembolic events while on anticoagulation medication. Echocardiography scheduled every three to six months should be considered to monitor the progression or resolution of the disease. 

Endocarditis and antiphospholipid syndrome (APS)

APS is characterized by recurrent venous or arterial thrombosis [7]. According to echocardiographic studies, approximately one-third of patients with primary APS had an increased prevalence of heart valve abnormalities [3]. Valvular disease, including thickening and vegetation, are the most common. Cardiac manifestations and disease have been observed in association with APS, including accelerated atherosclerosis, ventricular dysfunction, intracardiac thrombi and myxomas, and pulmonary hypertension. In APS, the presence of antiphospholipid antibodies such as anticardiolipin, anti-b2 glycoprotein, and lupus anticoagulant contribute to a procoagulant state [13]. 

APS can be classified as primary when it occurs as an isolated disorder and secondary when it presents along with another autoimmune disorder such as SLE. SLE patients with antiphospholipid antibodies (secondary APS) have increased morbidity and mortality as well as a higher frequency of valvular involvement than patients without these antibodies, as antiphospholipid antibodies promote thrombus formation on the endothelium of the injured valve. Valvular lesions linked with antiphospholipids present as valve masses or thickening, which may lead to valvular dysfunction. Regurgitation without stenosis is the most predominant functional abnormality, with the mitral valve being most affected [3].

Libman-Sacks endocarditis (a form of NBTE), which has been shown to be associated with SLE, can also be associated with APS [5]. This suggests that antiphospholipid antibodies may be involved in the pathogenesis of heart valve damage. In a study examining the immunopathologic findings in deformed heart valves of patients with primary and secondary APS, the researchers identified deposits of immunoglobulins (anticardiolipin antibodies [aCL]) and complement components [14]. 

Approximately 70 percent of APS patients have at least one valve abnormality diagnosed using echocardiography [15]. LSE has the most significant abnormality in the aortic and mitral valves. Diagnosing LSE usually requires the exclusion of infective endocarditis and rheumatic valve disease. Unless the patient is symptomatic, most physicians do not routinely screen for valvular lesions in patients with APS. If identified, valvular abnormalities require surgical intervention. LSE in APS patients is a strong risk factor for developing transient ischemic attack and stroke, which can increase mortality and morbidity in APS patients. As such, early detection, treatment of the underlying disease, and anticoagulation therapy reduce these risks for CV disease mortality in APS patients with NBTE [15].

The cornerstone therapy for most cardiac manifestations related to APS is anticoagulation therapy. Therapeutic guidelines for APS have aimed at either lowering blood hypercoagulability or antiphospholipid antibody levels [3]. Patients with definite APS with first venous thrombosis should be treated with warfarin at a target international normalized ratio (INR) of 2.0 to 3.0, and those at higher risk with recurrent events should be treated with warfarin at a target INR of greater than 3.0. Although there remain many challenges in preventing APS morbidity, anticoagulation has improved the morbidity and mortality associated with thrombotic APS. According to the Euro-Phospholipid cohort study of APS, researchers demonstrated a decrease in mortality from 5.3 percent in the initial five years of follow-up to 4.5 percent in the latter five years. In addition, treatment with anticoagulants in patients with obstetric APS had improved live births with an increase from 40 percent to 85 percent [16]. As a result, patients with asymptomatic elevated antiphospholipid antibody titers, pregnant women, and those with co-existent SLE should receive aspirin monotherapy [17]. It should be noted that the disease process and tissue injury are not amended by steroid treatment. Steroid therapy may lead to valve dysfunction by healing valvular vegetations, which results in scarring and valve deformity. Although valvular damage cannot be prevented with corticosteroid treatment, it may drastically suppress inflammatory reactions [3]. Further investigation into the role of antiphospholipid antibodies in the pathogenesis of valvular lesions might validate the use of targeted therapy. 

Diagnostics and management

Effective treatment of IE works to prevent and eliminate endocardial vegetation and secondary complications. Treatment with prolonged bactericidal antibiotics is utilized as well as surgical intervention, if necessary [18]. The duration and selection of antibiotic treatment are dependent on the resistance of the infecting bacteria and the nature of the involved valve. Severe bacterial infections should generally be treated using intravenous antibiotic therapy. Intravenous antibiotic therapy ensures a high level in blood as compared with orally administered antibiotics and is associated with more predictable pharmacokinetics. This approach allows for immediate action for sudden onset complications such as embolism. Meanwhile, an oral treatment for infective endocarditis has shown to be more effective than intravenous in patients with IE on the left side of their heart [19]. Antimicrobial treatments for infective endocarditis are advancing, and treatment regimens should be reviewed regularly. In addition, early surgical intervention is necessary in acute heart failure, extensive infection accompanied by localized complications, and recurrent arterial embolization.

Treatment of NBTE relies on the management of the underlying autoimmune disease. By treating the underlying mechanisms of these autoimmune diseases, including APS and SLE, with targeted approaches and management strategies, the risk of cardiovascular disease, including that caused by infective and NBTE endocarditis, may be reduced. The management of thrombosis in APS calls for antithrombotic therapies. Studies suggest that high intensity of anticoagulation therapy with an INR between 3.1 to 4.5 leads to a lower risk of recurrent thrombosis in patients with antiphospholipid (aPL) antibodies. In addition, patients with definite APS with first venous thrombosis should be treated with warfarin to an INR 2.0 to 3.0 and above 3.0 for those with recurrent and/or arterial events [17]. Treatment for patients with APS should be targeted to their presentation, as the condition is often difficult to assess since patients present with different etiologies and co-morbidities. Treatment recommendations for patients with SLE experiencing CV manifestations are based on the treatment of the underlying cardiac disease. The role of statins, antihypertensive drugs, aspirin, and lifestyle modifications are universally recognized and are essential in the management of SLE risk factors for CV disease. 

In a recent study of another autoimmune condition, researchers studied the effects of disease-modifying antirheumatic drug (DMARD) therapy on the effects of cardiovascular disease in patients with early rheumatoid arthritis (RA) [16,20]. RA is associated with an almost three-fold increase in mortality compared to the general population and is largely due to an increased association with cardiovascular disease (CVD). This higher incidence of mortality is largely due to an increased frequency of atherosclerosis and heart failure [21]. Some risk factors such as hypertension, smoking, high LDL-cholesterol level, and the risk of CVD in RA are mediated by inflammation. Shared immune mediators and mechanisms raise the possibility that RA therapies may also influence CVD pathogenesis. In one study, researchers evaluated whether early RA patients with treatment demonstrate any vascular changes compared to controls [2]. Patients who received etanercept and/or methotrexate had a lower incidence of CV events and improved vascular stiffness than those that stopped taking them [6,20]. The data from this study provides an impetus to consider anti-RA drugs as a treatment plan for autoimmune disease patients and shows how preventative pharmacotherapy use may help improve CV disease risk in patients. 

In addition to pharmacologic approaches, diagnostic monitoring may help to reduce cardiovascular risk in at-risk autoimmune patients, including those with SLE and APS. Researchers have found cardiovascular magnetic resonance (CMR) may be used to identify early signs of CV disease by assessing cardiac function and characterizing myocardial tissues in relation to edema and fibrosis. This effective diagnostic tool can be used as a preventative measure to evaluate acute rheumatic disease patients before clinical manifestations of heart disease that may later present [20]. 

The link between autoimmune diseases, IE, and oral bacterium has been studied and linked over the last couple of decades. As a result, it is well understood that both clinical practitioners and patients need to take a multi-disciplinary approach to management. This interdisciplinary approach, which includes working with healthcare workers in the dentistry field, can raise awareness and promote education to lower the risk of CV disease in autoimmune disease patients. With over 700 different bacterial species detected, the microbiota of the oral cavity is extremely diverse. There are certain sites in the mouth that have their own microbiota such as the cheek and the tongue. In 90 percent of cases, the microorganisms at fault are staphylococcus, viridans group streptococcus (*Streptococcus mutans* and *Streptococcus sanguinis*), and enterococcus [22]. Since they are components of dental plaque, they may enter the bloodstream inducing bacteremia through factors such as chewing or aggressive tooth brushing. In gingivitis, the infection is limited to the gingiva and reversible through proper oral hygiene [23]. When oral bacteria affect the surrounding tooth structures and tissue, this leads to periodontal infections, which are inflammatory diseases that involve immune-mediated responses and cannot be reversed but may be prevented. Specifically, periodontitis is capable of predisposing individuals to infective endocarditis, given the abundance of gram-negative bacteria involved, the levels of proinflammatory cytokines, and the heavy inflammatory infiltrates involved [24].

**Figure 2:**
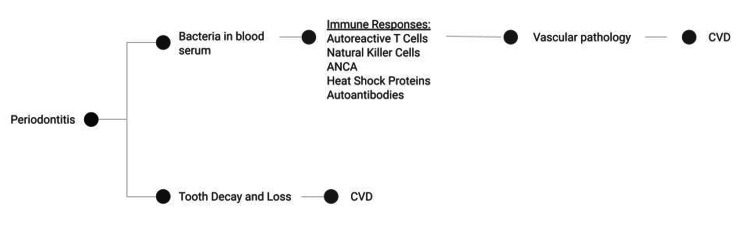
Proposed mechanisms linking oral infection and periodontal disease to cardiovascular disease CVD - cardiovascular disease

Represented in Figure 2 is the pathogenesis of periodontitis, which involves immune responses from autoreactive T cells, natural killer cells, anti-neutrophil cytoplasmic antibodies (ANCA), heat shock proteins, autoantibodies, and genetic factors to bacterial antigens of dental plaque that accumulate on teeth and associated tissue [25]. ANCAs constitute antibodies that target antigens present in azurophil granules of polymorphonuclear leukocytes (PMNs). Various studies have proven the role of ANCA in several autoimmune and inflammatory diseases as well as its role in the pathogenesis of periodontal disease through activation of cells with ANCA antigens that lead to inflammatory responses and results in bystander damage to cells with the target antigens [26]. Factors that also place patients at a high risk for periodontal disease may also place them at a high risk for systemic diseases, such as cardiovascular disease. Early detection and effective treatment for periodontal infections are critical in reducing bacteremia and preventing cases of IE [27].

## Conclusions

Overall, a higher risk of CV disease complications, including elevated CV morbidity and mortality, is associated with infective and/or NBTE endocarditis in patients with autoimmune diseases such as SLE and APS. The underlying mechanism of disease activity associated with these autoimmune conditions weakens the cardiovascular system components, leaving patients more susceptible to inflammation and infectious agents. While diagnosis and management can vary based on underlying etiology, an interdisciplinary approach that includes prevention and management from dentists, cardiologists, rheumatologists, and primary care physicians is needed. In addition, increasing patient and physician education on risk factors and prevention strategies is highly recommended. Working in collaboration, practicing clinicians can ensure an optimal treatment in immune-deficient patients and reduce the risk of adverse cardiovascular outcomes. Implementing an early screening, management, and treatment plan in a targeted approach to patients with autoimmune diseases may better prevent the onset of heart disease and improve survival outcomes for these patients.
